# The Composition Analysis of Renal Staghorn Calculi and their Characteristics using Spectral CT

**DOI:** 10.2174/0115734056389587250417055946

**Published:** 2025-06-13

**Authors:** Xian Li, Qiao Zou, Lili Ou, Lilan Chen, Jingming Wang, Xinchun LI

**Affiliations:** 1 The Department of Radiology, the First Affiliated Hospital of Guangzhou Medical University, Guangdong, Guangzhou, People’s Republic of China; 2 The Guangdong Key Laboratory of Urology, the First Affiliated Hospital of Guangzhou Medical University, Minimally Invasive Surgery Center, Guangdong, Guangzhou 510120, People’s Republic of China; 3 The Department of Radiology, Zhuhai People’s Hospital, Zhuhai Hospital Affiliated of Jinan University, Zhuhai, People’s Republic of China; £ Present Address: The Department of Radiology, the First Affiliated Hospital of Guangzhou Medical University, Guangdong, Guangzhou51020, People’s Republic of China

**Keywords:** Computed tomography, Dual energy, Kidney, Staghorn calculi, Composition, *In vivo*, Zeff values, Spectroscopy

## Abstract

**Objective:**

This study aimed to analyze the composition of renal staghorn calculi and their characteristics using spectral CT.

**Methods:**

This study enrolled 111 cases of renal staghorn calculi from 94 patients (48 males and 46 females, aged 28–76 years; median age: 56 years). Using spectral CT, average Zeff and CT values were analyzed. The water/iodine-based images were generated by the material separation module. All stones were detected by FTIR spectroscopy.

**Results:**

111 cases of renal staghorn calculi included 53 cases of single composition (47.8%) and 58 cases of mixed composition (52.2%). In staghorn calculi of a single composition, urate (23 cases) and calcium oxalate monohydrate (16 cases) were more prevalent than struvite (5 cases) and brushite (5 cases). Mixed compositions included metabolic-metabolic (36 cases, 62.1%), metabolic-infectious (14 cases, 24.1%), and infectious-infectious (8 cases, 13.8%) cases, respectively. The average Zeff values showed some characteristics of carbapatite and urate. However, average Zeff and CT values had many overlappings among other compositions. All stones appeared homogeneous in water-based images. In iodine-based images, calcium oxalate monohydrate displayed homogeneous high density, but struvite and brushite showed heterogeneous high density. Single compositions of carbapatite, calcium oxalate monohydrate, and cystine exhibited homogeneous high density, similar to mixed compositions of carbapatite and calcium oxalate monohydrate. Furthermore, urate demonstrated homogeneous low density. Moreover, the mixture of struvite and brushite/urate showed heterogeneous high density.

**Conclusion:**

In staghorn calculi of a single composition, the metabolic type was common, while metabolic-metabolic and metabolic-infectious types frequently occurred in staghorn calculi with mixed compositions. Except for average Zeff values, water-iodine material separation performed an important auxiliary function in differentiating stones’ compositions using spectral CT.

## 1 INTRODUCTION

Rassweiler JJ described renal staghorn calculi as branched shape, which could be categorized into four types, including borderline, partial, complete, and gigantic [1]. Based on the extension of staghorn calculi, the borderline stones occupy the renal pelvis and one calyx, the partial stones extend to the renal pelvis and two calyces, the complete stones involve the renal pelvis and over 80% of all calyces, and the gigantic stones encompass the entire renal collecting system. Due to the large volume of staghorn calculi, patients with renal staghorn calculi often present with hydronephrosis, renal function decline, and renal failure [2]. Mishra *et al*. reported the standard treatment for staghorn calculi to be percutaneous nephrolithotomy (PCNL), which effectively removes these complex stones and significantly reduces the burden on the renal collecting system [3]. Moreover, ureteroscopy could be used to eliminate the residual stones [4].

Traditionally, struvite is considered to account for a large proportion of staghorn calculi, characterized by a high content of organic matrix [5]. Due to high fragility, renal staghorn calculi with the composition of struvite could be easy to remove. However, Akif Diri suggested that metabolic stones constitute a higher proportion than struvite in staghorn calculi [6]. This trend may be attributed to the changes in dietary and lifestyle habits, as well as the rising prevalence of metabolic diseases, which are associated with increased levels of urate and calcium oxalate [7]. The other contributing factors include urinary tract obstruction, anatomical abnormalities, and prolonged use of indwelling catheters [8].

Renal staghorn calculi may be examined by ultrasound, kidney-ureter-bladder (KUB) radiography, and intravenous pyelography (IVP). Compared to general computed tomography (CT), these conventional techniques may exhibit inherent limitations in the appearance of staghorn calculi [9, 10]. Ultrasound is usually used as the primary imaging modality to detect renal staghorn calculi. However, dual-energy CT could enable quantitative differentiation between uric acid and non-uric acid stones, having a critical advantage over general single-energy CT [11]. Moreover, the spectral CT (rapid kV switching dual-energy CT) has demonstrated significant potential ability to improve the diagnostic accuracy of stones’ compositions, especially the non-uric acid stones and mixed stones [12]. The Zeff value and the water/iodine-based images could be used to analyze compositions of staghorn calculi [13].

In this study, the compositions of single and mixed staghorn calculi were detected by Fourier transform infrared (FTIR) spectroscopy, which can be easily adopted in routine laboratories and provide accurate results [14]. Then, the characteristics of renal staghorn calculi were analyzed using spectral CT based on the results of FTIR.

## 2 MATERIALS AND METHODS

### 2.1 Patients and Stones

This retrospective study included 111 cases of renal staghorn calculi from 94 patients, of which 18 patients had two renal stones. These patients included 48 males and 46 females (aged 28–76 years, median: 56 years), who were recruited from January 1st, 2019, to January 31st, 2023. Common presenting symptoms of these patients included dysuria, hematuria, urinary retention, and pain. After examination by spectral CT (Revolution, GE Healthcare), all patients underwent percutaneous nephrolithotomy (PCNL). All removed stones were analyzed using FTIR spectroscopy to define their compositions.

### 2.2 CT Analysis

In the spectral CT examination, the gemstone spectral imaging (GSI) modes were chosen as the scanning modes. Non-contrast CT scanning was used by employing the following parameters: instantaneous switching of tube voltage from 80 kVp to 140 kVp, the tube current of 260 mA, rotating speed of 0.7 s/R, slice thickness of 0.625 mm, and pitch of 0.984. The data obtained were subjected to the post-processing workstation for reconstruction.

### 2.3 Reconstruction and Parameter Analysis

By employing the AW 4.7 post-processing workstation, the average Zeff values were analyzed using the atomic number module, while the average CT values were detected using a 70 keV virtual mono-energetic image. The data were analyzed three times, and the intermediate value was used. Secondly, water-based images and iodine-based images were observed by the material separation module. For every stone, the transverse and coronal images were observed in three slices to acquire appropriate images. It was predicted that these appropriate images should show the maximum dimension of renal staghorn calculi and assess the entire situation of renal staghorn calculi. Finally, these images were observed and detected carefully. The white color represented the high density, while the black color represented the low density.

### 2.4 Statistical Analysis

SPSS 19.0 (SPSS, Inc., Chicago, IL, USA) was employed for statistical analysis. The average Zeff values and average CT values among different groups were statistically analyzed by using one-way analysis of variance (ANOVA). Statistical difference was considered at a P-value less than 0.05.

## 3 RESULTS

### 3.1 Types of Renal Staghorn Calculi

111 cases of renal staghorn calculi from 94 patients were examined, including 53 cases (47.8%) with single composition and 58 cases (52.2%) with mixed compositions (Table **1**). Notably, 3 patients exhibited two renal stones with different compositions, whereas the other 15 patients had two renal stones with the same compositions.

Groups 1-6 included stones with single compositions (53 cases), corresponding to carbapatite (3 cases), struvite (5 cases), brushite (5 cases), urate (23 cases), calcium oxalate monohydrate (16 cases), and cystine (1 case). In staghorn calculi with a single composition, the cases of urate and calcium oxalate monohydrate were more than the cases of struvite and brushite. However, carbapatite and cystine were relatively rare, and only a limited number of cases were observed.

As presented in Table **1**, groups 7-17 comprised the renal staghorn calculi with mixed compositions (58 cases), which were classified into metabolic-metabolic type, metabolic-infectious type, and infectious-infectious type. For the metabolic-metabolic type, the cases of calcium oxalate monohydrate and calcium oxalate dihydrate were the most prevalent (19 cases). The secondary cases included calcium oxalate monohydrate and urate (11 cases). In the other cases of mixed metabolic compositions, the metabolic-metabolic type accounted for a total of 36 cases. There were 8 cases of infectious-infectious stones with mixed compositions of struvite and brushite. However, 14 cases of renal staghorn calculi had the metabolic and infectious compositions at the same time, including 8 cases of calcium oxalate monohydrate and carbapatite, 1 case of calcium oxalate monohydrate and struvite, and 5 cases of calcium oxalate monohydrate and brushite.

### 3.2 Characteristics of Renal Staghorn Calculi with Single Composition on Spectral CT

For renal staghorn calculi with a single composition (Table **2**), the average Zeff values of carbapatite (13.99-14.17) and urate (7.10-10.05) exhibited special characteristics. However, there were overlappings of the average Zeff values between calcium oxalate monohydrate and struvite/brushite. Moreover, only carbapatite had a special average CT value (1447-1544 HU). The average CT values of other compositions were non-specific, being unsuitable for accurate diagnosis. Cystine accounted for only 1 case, whose Zeff value and average CT value were 11.23 and 736 HU, respectively.

To increase the diagnostic accuracy of various compositions, the iodine-based and water-based images could show auxiliary advantages, especially the iodine-based images (Table **3**). In iodine-based images, calcium oxalate monohydrate displayed a homogeneous high density, whereas struvite and brushite showed a heterogeneous high density (Figs. **1** and **2**). The iodine-based images and the average Zeff values could be analyzed together to increase the diagnostic accuracy. Carbapatite showed a homogeneous high density in iodine-based images, and urate showed a homogeneous low density in iodine-based images (Figs. **3** and **4**). All renal staghorn calculi exhibited homogeneous high densities in water-based images.

### 3.3 Characteristics of Renal Staghorn Calculi with Mixed Compositions on Spectral CT

In the case of staghorn calculi with mixed compositions, there were no special characteristics among different stones due to many overlappings between average Zeff values and average CT values. For example, the average Zeff values of stones having mixed compositions of struvite and brushite were 12.55 ± a0.42, while the average Zeff values of stones with mixed compositions of urate and calcium oxalate monohydrate were 12.29 ± i0.624, respectively. There was no statistical difference between two different mixed stones (P = 0.09, > 0.05). The average CT values were also inadequate for accurate diagnosis. The average CT values of stones with mixed compositions of struvite and brushite were 896.13 ±e145.406 HU, while the average CT values of stones with mixed compositions of urate and calcium oxalate monohydrate were 904.73 ±0165.702 HU, respectively. There was no statistical difference found between these two different mixed stones (P = 0.933, > 0.05).

Table **3** shows that all staghorn calculi with mixed compositions of carbapatite and calcium oxalate monohydrate exhibited homogeneous high density in the iodine-based images (Fig. **5**). Moreover, staghorn calculi with mixed compositions of struvite and brushite showed heterogeneous high density. In the case of staghorn calculi involving mixed compositions of urate and other stones, heterogeneous low density could be observed (Fig. **6**).

## 4 DISCUSSION

### 4.1 Types of Renal Staghorn Calculi

According to etiology, renal staghorn calculi could be classified into four categories, including infectious stones (comprising struvite, carbapatite, and brushite), metabolic stones (including calcium oxalate, urate, and cystine), stones associated with genetic defects, and stones caused by adverse drug effects (drug-induced stones) [15]. In this study on staghorn calculi with a single composition, the cases of typical metabolic stones (specifically urate and calcium oxalate monohydrate) were significantly more than the cases of typical infectious stones (primarily struvite and brushite). This result showed metabolic staghorn calculi to have a higher incidence than infectious ones in recent years, being different from the traditional concept (staghorn calculi caused by infection) [16]. In the case of staghorn calculi with mixed compositions, this study observed a high incidence of mixed metabolic stones (62.07%), especially calcium oxalate monohydrate mixed with calcium oxalate dihydrate and calcium oxalate monohydrate mixed with urate. Similarly, a research study from Sri Lanka also showed calcium oxalate as the predominant composition of renal staghorn calculi, and infection stones only occupied 10% of staghorn calculi [17]. This distribution pattern was further supported by Viprakasit's report, which documented 56% of the cases of renal staghorn calculi to comprise metabolic stones [18].

Furthermore, the infectious composition of staghorn calculi could be mixed with infectious composition or metabolic composition. Specifically, we identified three predominant mixed patterns, including struvite combined with brushite and struvite/brushite combined with calcium oxalate. Additionally, the frequently observed combination was calcium oxalate monohydrate mixed with carbapatite, suggesting potential metabolic-infectious interactions in the formation of staghorn calculi. The incidence of metabolic-metabolic staghorn calculi was the highest, followed by metabolic-infectious ones. They occurred more frequently than infectious-infectious staghorn calculi. This finding has been found to be consistent with previous literature, which has also reported a higher prevalence of mixed metabolic-metabolic and metabolic-infectious stones [19]. As metabolic staghorn calculi have been more prevalent than infectious staghorn calculi in recent studies, metabolic evaluation has been deemed necessary to prevent metabolic staghorn calculi [18]. Common metabolic abnormalities have been reported to be hypercalciuria, hypocitraturia, and hypernatriuria [2]. These patients usually exhibit metabolic abnormalities and should undergo 24-hour urine testing, serum calcium level measurement, comprehensive urinalysis, and urine culture.

### 4.2 Advantages of Spectral CT

Spectral CT could provide comprehensive visualization of renal staghorn calculi, including the location and size of stones, the skin-to-stone distance, and the relation between stones and adjacent organs [20]. Furthermore, this advanced image modality enables precise quantification, including volume and density measurements, the angles between the entry point and stone branches, and the infundibular neck width at the calyceal entry site [21-23].

Moreover, spectral CT offers some important parameters for composition analysis, such as Zeff value and material separation. When an element and a compound have the same attenuation of X-rays, the Zeff value of this element could be used to represent the Zeff value of this compound [24]. The Zeff value exhibits important diagnostic significance, as it offers specific characteristics for material differentiation. The spectral CT demonstrated a unique capability to convert conventional attenuation data into distinct material-specific measurements by using basis-pair decomposition. So the X-ray absorption of one material (even the complex compound or structure) could be expressed by the absorption of other substances. According to the images of different material densities, this technique allowed for detailed visualization of the stone’s composition. In clinical practice, water-based images and iodine-based images are typically employed as the standard basis-pair materials, providing a reliable framework for composition analysis [25].

### 4.3 Characteristics of Renal Staghorn Calculi on Spectral CT

For renal staghorn calculi with a single composition in this study, the average Zeff values exhibited special characteristics for carbapatite (13.99-14.17) and urate (7.10-10.05). However, the average Zeff values demonstrated limited diagnostic utility for calcium oxalate monohydrate and struvite/brushite stones. Furthermore, the average Zeff values were not suitable for staghorn calculi with mixed compositions in this study. Compared to those with single composition, mixed-composition staghorn calculi have been found to exhibit a higher prevalence in China and other regions of the world [26, 27]. As the average Zeff value is calculated based on the various compositions and their proportions, it may be difficult to analyze staghorn calculi solely using the average Zeff value.

Moreover, the average CT value of carbapatite showed to range from 1447 to 1544 HU. However, the average CT values had many overlappings among different stones with single or mixed compositions. Notably, the discriminative power of the average CT value has been found to be more useful than that of the average Zeff value as the CT value is influenced by multiple factors, including material density, thickness, and the relative proportions of various compositions. Also, some literature has demonstrated the potential utility of average CT values in clinical practice, particularly in predicting stone fragility through average CT values and their standard deviations [28].

The material separation function could analyze the content of two specific basic substances accurately [29]. In our study, iodine-based and water-based images were used to achieve auxiliary advantages and improve the diagnostic accuracy of various staghorn calculi. Although the average Zeff values of calcium oxalate monohydrate and struvite/brushite had some overlapping, they displayed homogeneous high density or heterogeneous high density in iodine-based images. The renal staghorn calculi of struvite and brushite showed heterogeneous high density both in single or mixed stones, as also observed in the literature [30]. Furthermore, special characteristics were observed for carbapatite and urate stones. Carbapatite demonstrated a homogeneous high density, while urate consistently showed a homogeneous low density in iodine-based images. The combined analysis of iodine/water-based images and average Zeff values significantly enhanced the diagnostic accuracy of staghorn calculi with a single composition, providing a comprehensive approach to stone characterization.

For staghorn calculi with mixed composition, the mixture of carbapatite and calcium oxalate monohydrate exhibited a homogeneous high density in the iodine-based images. The homogeneous high density meant that the compositions of urate, struvite, and brushite could not exist in staghorn calculi with mixed compositions. Some literature studies have only observed general renal stones, but they have reported similar results [29, 31].

Furthermore, all staghorn calculi exhibited homogeneous high densities in the water-based images in our study, including uric acid and non-uric acid stones. Similarly, the non-uric acid stones showed high density in iodine-based and water-based images, but uric acid stones only appeared in water-based images [32]. Roberto C also established that water/iodine-based separation images could distinguish uric acid stones and non-uric acid stones [33].

### 4.4 Limitation

Some limitations of this study should be discussed. Although struvite and brushite were both infectious stones, it was difficult to differentiate struvite and brushite by Zeff values and the iodine-based images. In staghorn calculi with mixed compositions, the mixture of urate and other compositions was similar to the mixture of struvite and brushite according to Zeff values. These diagnostic challenges highlighted the need for the development of advanced analytical approaches to improve accuracy.

Additionally, it is important to note that the material decomposition images utilized in spectral CT were generated through computer post-processing algorithms rather than direct scanning. The accuracy of material separation may have been decreased in obese patients due to the noisy images resulting from low X-ray energy. To enhance the reliability of our findings, future studies should incorporate a large number of cases, preferably through multi-center research. Furthermore, comprehensive analysis should integrate clinical parameters (including age and gender distribution) and laboratory data (such as complete blood count, uric acid, citric acid, calcium, and phosphorus levels) to improve diagnostic accuracy.

## CONCLUSION

In our analysis of staghorn calculi with a single composition, metabolic stones (including urate and calcium oxalate monohydrate) demonstrated a higher prevalence compared to infectious stones (including struvite and brushite). Furthermore, staghorn calculi with mixed compositions predominantly consisted of metabolic-metabolic and metabolic-infectious combinations. Except for the diagnostic utility of average Zeff values, the water/iodine-based material decomposition provided significant value in differentiating various stones by *in vivo* spectral CT. This dual-parameter diagnostic method enhanced diagnostic accuracy by combining specific material density with atomic composition analysis.

## AUTHORS’ CONTRIBUTIONS

XL: Conceptualized the study; JW: Provided administrative support; XL and QZ: Provided study materials and recruited patients; LC: Contributed to the collection and assembling of data; LO: Performed data analysis and interpretation.

## Figures and Tables

**Fig. (1a):**
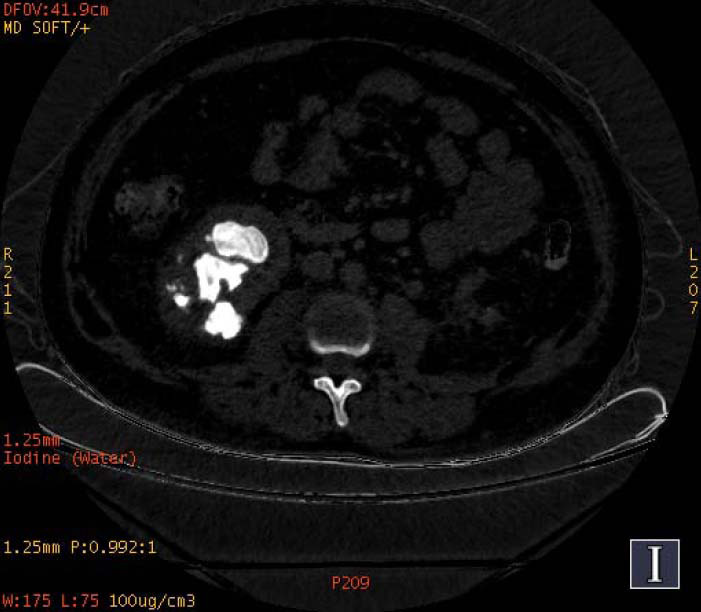
The staghorn calculi of struvite in the right kidney exhibited heterogeneous high density in iodine-based images, in which a large low-density area could also be observed.

**Fig. (1b):**
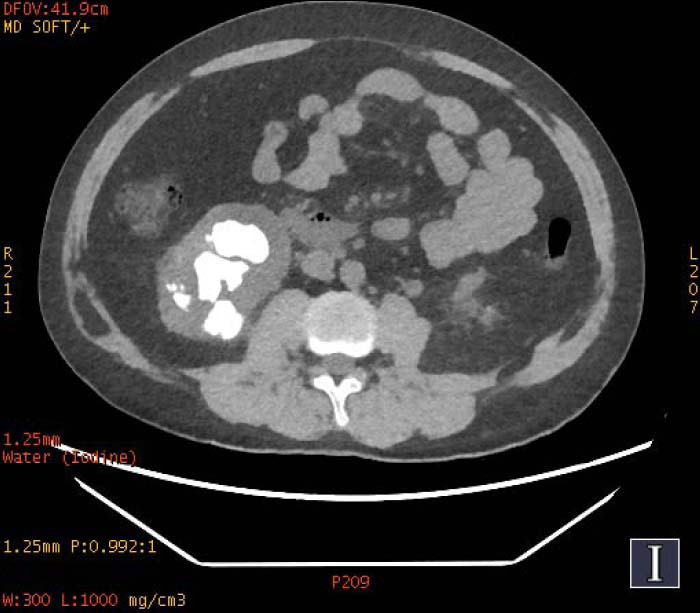
The staghorn calculi of struvite in the right kidney exhibited homogeneous high density in water-based images.

**Fig. (2a):**
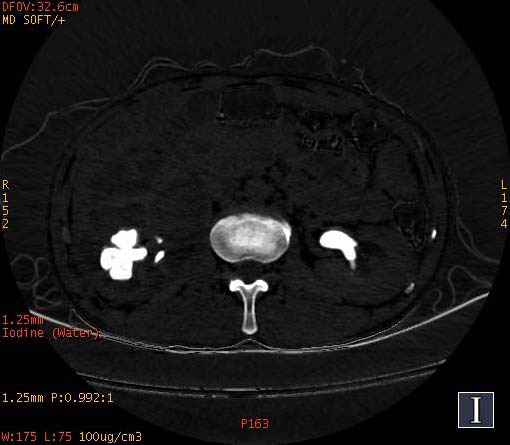
In iodine-based images, the staghorn calculi of brushite in the right kidney exhibited heterogeneous high-density, with a small low-density area. The staghorn calculi of calcium oxalate monohydrate in the left kidney exhibited homogeneous high density.

**Fig. (2b):**
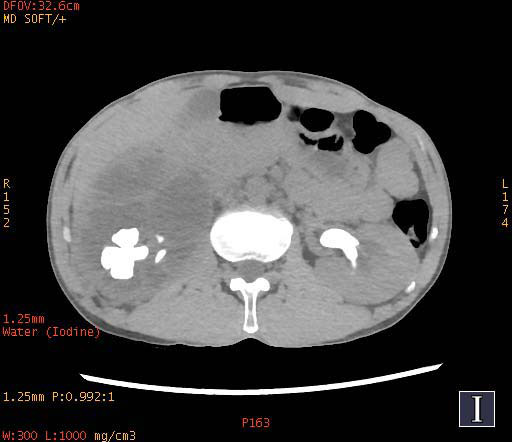
In water-based images, the staghorn calculi of brushite in the right kidney and the staghorn calculi of calcium oxalate monohydrate in the left kidney demonstrated homogeneous high density.

**Fig. (3a):**
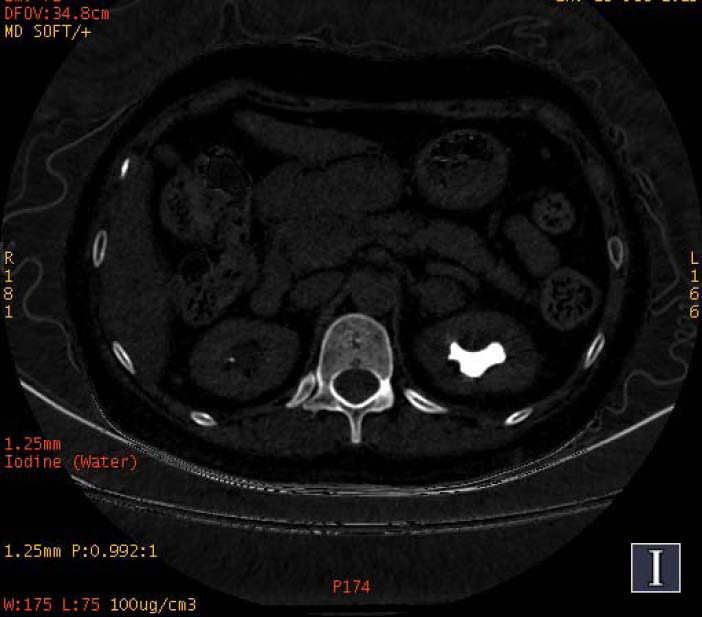
The staghorn calculi of carbapatite in the left kidney exhibited homogeneous high density in iodine-based images.

**Fig. (3b):**
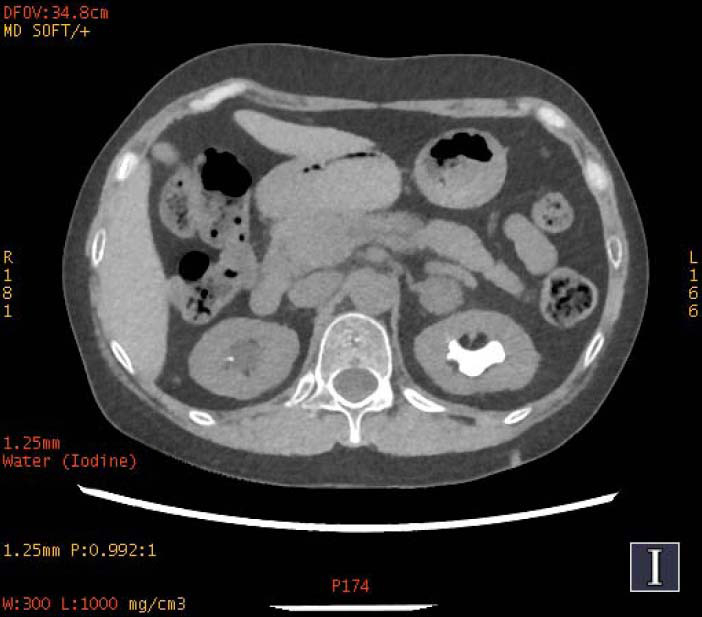
The staghorn calculi of carbapatite in the left kidney exhibited homogeneous high density in water-based images.

**Fig. (4a):**
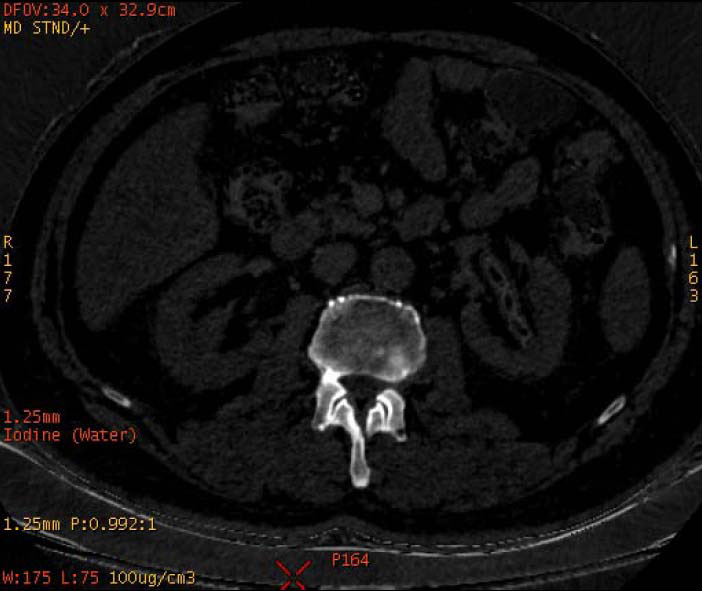
The staghorn calculi of urate in the left kidney exhibited homogeneous low density in iodine-based images.

**Fig. (4b):**
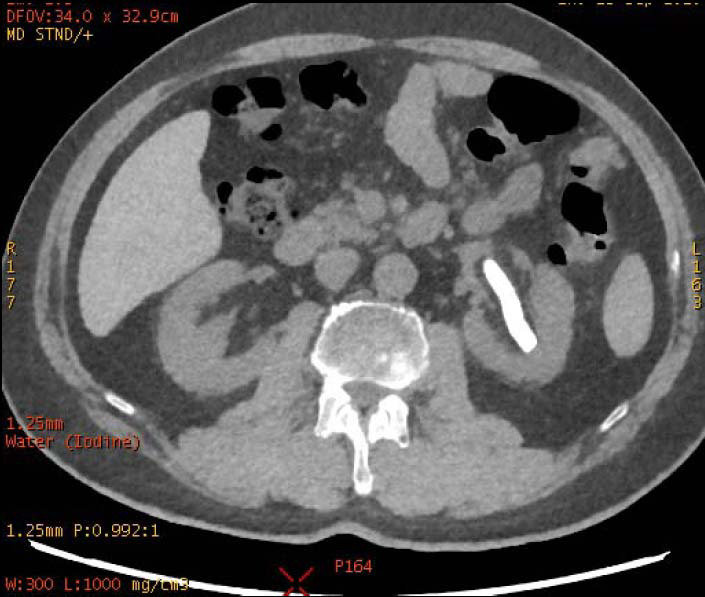
The staghorn calculi of urate in the left kidney exhibited homogeneous high density in water-based images.

**Fig. (5a):**
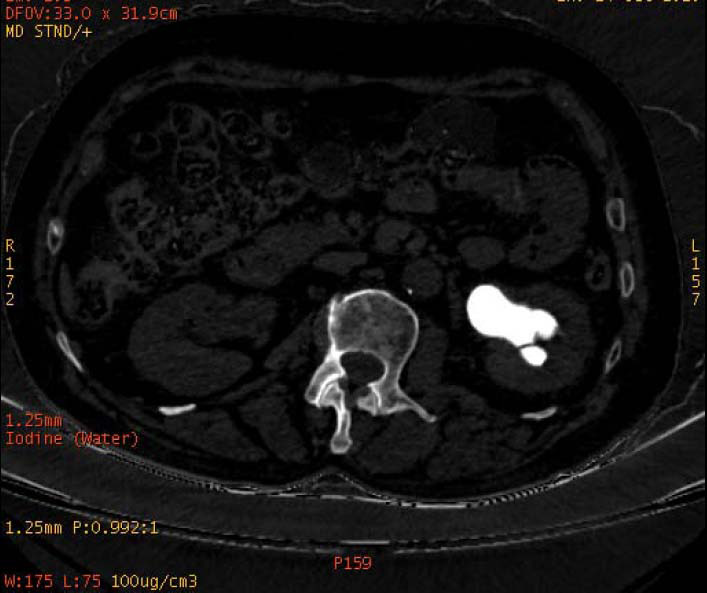
The staghorn calculi with a mixed composition of carbapatite and calcium oxalate monohydrate in the left kidney exhibited homogeneous high density in iodine-based images.

**Fig. (5b):**
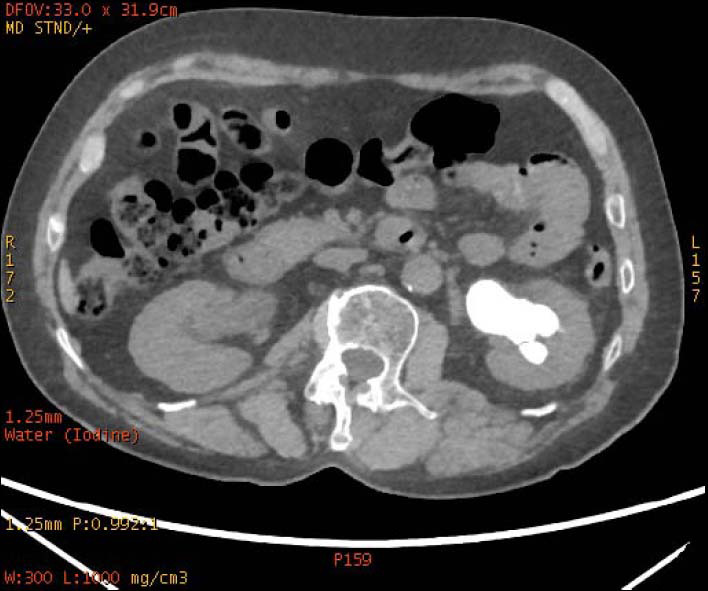
The staghorn calculi with a mixed composition of carbapatite and calcium oxalate monohydrate in the left kidney demonstrated homogeneous high density in water-based images.

**Fig. (6a):**
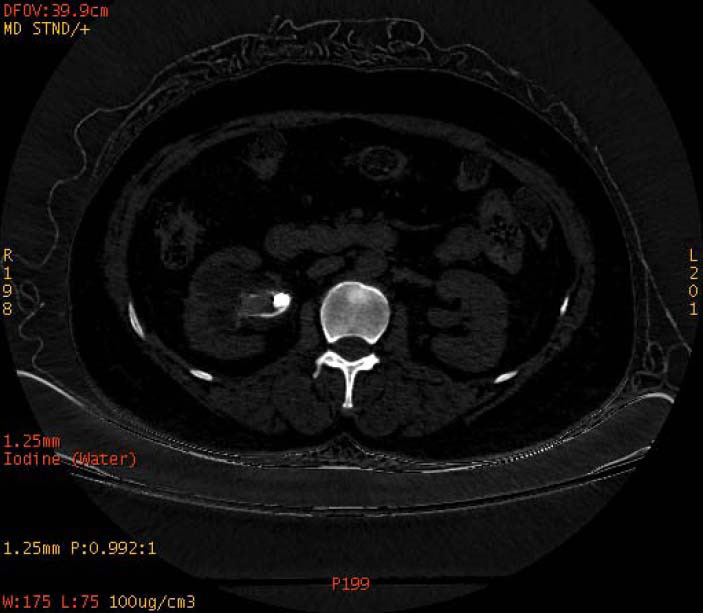
The staghorn calculi with a mixed composition of urate and carbapatite in the right kidney exhibited heterogeneous low density in iodine-based images.

**Fig. (6b):**
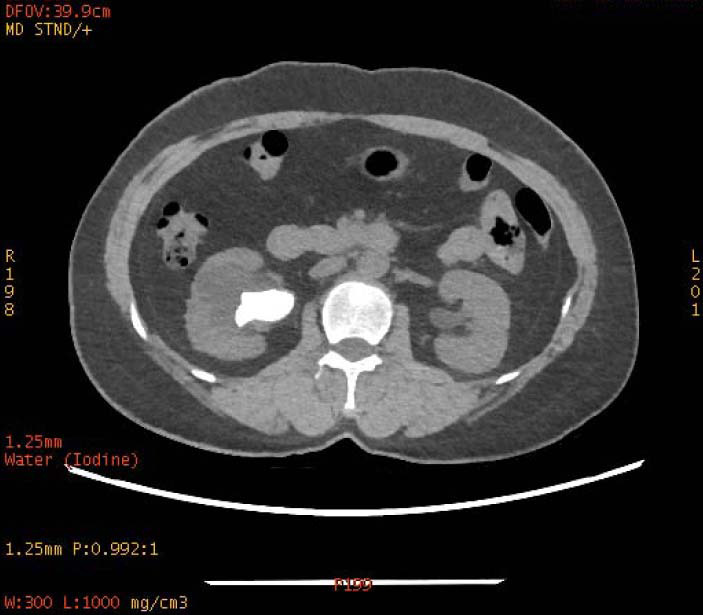
The staghorn calculi with a mixed composition of urate and carbapatite in the right kidney demonstrated homogeneous high density in water-based images.

**Table 1:** The compositions of renal staghorn calculi.

Composition	Carbapatite	Struvite	Brushite	Urate	Calcium Oxalate Monohydrate	Calcium Oxalate Dihydrate	Cystine	Numbers	Percentage
Group 1	√							3 cases	2.70%
Group 2		√						5 cases	4.50%
Group 3			√					5 cases	4.50%
Group 4				√				23 cases	20.72%
Group 5					√			16 cases	14.41%
Group 6							√	1 case	0.90%
Group 7	√	√						2 cases	1.80%
Group 8	√		√					1 case	0.90%
Group 9	√			√				1 case	0.90%
Group 10	√				√			8 cases	7.21%
Group 11	√					√		1 case	0.90%
Group 12		√	√					8 cases	7.21%
Group 13		√			√			1 case	0.90%
Group 14			√		√			4 cases	3.60%
Group 15				√	√			11 cases	9.91%
Group 16				√		√		2 cases	1.80%
Group 17					√	√		19 cases	17.12%
Total								111 cases	100%

**Table 2:** The ranges of average Z_eff_ values and the average CT values in different renal staghorn calculi.

	Carbapatite	Struvite	Brushite	Urate	Calcium Oxalate Monohydrate	Cystine
The average Zeff value	13.99-14.17	11.78-12.75	12.79-13.28	7.10-10.05	12.48-13.80	11.23
The average CT value	1447-1544 HU	605-1198 HU	919-1263 HU	314-815 HU	778-1391 HU	736 HU

**Table 3:** The densities of water-based and iodine-based images for renal staghorn calculi.

	Group 1	Group 2	Group 3	Group 4	Group 5	Group 6	Group 7	Group 8	Group 9	Group 10	Group 11	Group 12	Group 13	Group 14	Group 15	Group 16	Group 17
Homogeneous high density (water-based images)	3	5	5	23	16	1	2	1	1	8	1	8	1	4	11	2	19
Homogeneous high density (iodine-based images)	3				16	1				8	1						19
Homogeneous low density (iodine-based images)				23													
Heterogeneous high density (iodine-based images)		5	5				2	1	1			8	1	4	11	2	
Numbers	3 cases	5 cases	5 cases	23 cases	16 cases	1 case	2 cases	1 case	1 case	8 cases	1 case	8 cases	1 case	4 cases	11 cases	2 cases	19 cases

*Each case has one sequence of water-based images and one sequence of iodine-based images.

## Data Availability

The data of current study are available from corresponding authors [J.W] and [X.L], on a reasonable request.

## LIST OF ABBREVIATIONS

CT: Computed tomography
Spectral CT: Rapid kV switching dual-energy CT
FTIR: Fourier transform infrared
GSI: Gemstone spectral imaging
ANOVA: Analysis of variance
PCNL: Percutaneous nephrolithotomy

## STANDARDS OF REPORTING

STROBE guidelines were followed.
